# Optimal clustering under uncertainty

**DOI:** 10.1371/journal.pone.0204627

**Published:** 2018-10-02

**Authors:** Lori A. Dalton, Marco E. Benalcázar, Edward R. Dougherty

**Affiliations:** 1 Department of Electrical and Computer Engineering, The Ohio State University, Columbus, OH, United States of America; 2 Departamento de Informática y Ciencias de la Computación, Facultad de Ingeniería de Sistemas, Escuela Politécnica Nacional, Quito, Ecuador; 3 Department of Electrical and Computer Engineering, Texas A&M University, College Station, TX, United States of America; Tampere University of Technology, FINLAND

## Abstract

Classical clustering algorithms typically either lack an underlying probability framework to make them predictive or focus on parameter estimation rather than defining and minimizing a notion of error. Recent work addresses these issues by developing a probabilistic framework based on the theory of random labeled point processes and characterizing a *Bayes clusterer* that minimizes the number of misclustered points. The Bayes clusterer is analogous to the Bayes classifier. Whereas determining a Bayes classifier requires full knowledge of the feature-label distribution, deriving a Bayes clusterer requires full knowledge of the point process. When uncertain of the point process, one would like to find a robust clusterer that is optimal over the uncertainty, just as one may find optimal robust classifiers with uncertain feature-label distributions. Herein, we derive an optimal robust clusterer by first finding an *effective* random point process that incorporates all randomness within its own probabilistic structure and from which a Bayes clusterer can be derived that provides an optimal robust clusterer relative to the uncertainty. This is analogous to the use of effective class-conditional distributions in robust classification. After evaluating the performance of robust clusterers in synthetic mixtures of Gaussians models, we apply the framework to granular imaging, where we make use of the asymptotic granulometric moment theory for granular images to relate robust clustering theory to the application.

## Introduction

The basic optimization paradigm for operator design consists of four parts: (1) define the underlying random process; (2) define the class of potential operators; (3) characterize operator performance via a cost function; and (4) find an operator to minimize the cost function. The classic example is the Wiener filter, where the four parts consist of wide-sense stationary true and observed signals, linear operators, minimization of the mean-square error, and optimization in terms of power spectra. In practice, we might be uncertain as to the distribution governing the random process so that we desire a *robust* operator, one whose performance is acceptable relative to the uncertainty. Robust design can be posed in the following way: Given a class of operators and given that the *state of nature* is uncertain but contained in some *uncertainty class*, which operator should be selected to optimize performance across all possible states of nature?

Our interest here is in clustering, where the underlying process is a random point set and the aim is to partition the point set into clusters corresponding to the manner in which the points have been generated by the underlying process. Having developed the theory of optimal clustering in the context of random labeled point sets where optimality is with respect to mis-clustered points [1], we now consider optimal clustering when the underlying random labeled point process belongs to an uncertainty class of random labeled point processes, so that optimization is relative to both clustering error and model uncertainty. This is analogous to finding an optimal Wiener filter when the signal process is unknown, so that the power spectra belong to an uncertainty class [2]. We now briefly review classical robust operator theory, which will serve as the foundation for a new general theory of optimal robust clustering.

Optimal robust filtering first appeared in signal processing in the 1970s when the problem was addressed for signals with uncertain power spectra. Early work considered robust filter design from a minimax perspective: the filter is designed for the state having the best worst-case performance over all states [3–5]. Whereas the standard optimization problem given certainty with regard to the random process takes the form

$$\begin{array}{c} \psi^{*}=\text{arg}\;\underset{\psi \in C}{\text{min}}\;\gamma \left(\psi \right), \end{array}$$ (1)

where C is the operator class and *γ*(*ψ*) is the cost of applying operator *ψ* on the model, minimax optimization is defined by

$$\begin{array}{c} \psi_{\text{MM}}=\text{arg}\;\underset{\psi \in C_{\Theta }}{\text{min}}\underset{\theta \in \Theta }{\text{max}}\;\gamma_{\theta }\left(\psi \right), \end{array}$$ (2)

where Θ is the uncertainty class of random processes, $C_{\Theta }$ is the class of operators that are optimal for some state in the uncertainty class, and *γ*_*θ*_(*ψ*) is the cost of applying operator *ψ* for state *θ* ∈ Θ.

Suppose one has prior knowledge with which to construct a prior distribution *π*(*θ*) on states (models) in the uncertainty class. Rather than apply a minimax robust operator, whose average performance can be poor, a Bayesian approach can be taken whereby optimization is relative to *π*(*θ*). A *model-constrained (state-constrained) Bayesian robust (MCBR) operator* minimizes the expected error over the uncertainty class among all operators in $C_{\Theta }$:

$$\begin{array}{c} \psi_{\text{MCBR}}=\text{arg}\;\underset{\psi \in C_{\Theta }}{\text{min}}E_{\theta }\left[\gamma_{\theta }\left(\psi \right)\right]. \end{array}$$ (3)

MCBR filtering has been considered for morphological [6], binary [7] and linear [8] filtering. MCBR design has also been applied in classification with uncertain feature-label distributions [9].

Rather than restrict optimization to operators that are optimal for some state in the uncertainty class, one can optimize over any class of operators, including unconstrained optimization over all possible measurable functions. In this case, the optimal operator is called an *intrinsically optimal Bayesian robust (IBR) operator (filter)* and Eq (3) becomes

$$\begin{array}{c} \psi_{\text{IBR}}=\text{arg}\;\underset{\psi \in C}{\text{min}}\;E_{\theta }\left[\gamma_{\theta }\left(\psi \right)\right], \end{array}$$ (4)

where C is a set of operators under consideration. IBR filtering has been considered for linear and morphological filtering [2]. The IBR approach was first used to design optimal classifiers when the unknown true feature-label distribution belongs to an uncertainty class [10, 11]. In that setting, optimization is relative to a posterior distribution obtained from the prior utilizing sample data and an optimal classifier is called an *optimal Bayesian classifier (OBC)*.

Unlike the state of affairs in filtering and classification, classical clustering algorithms typically lack an underlying probability framework to make them predictive. The exceptions, for instance, expectation-maximization based on mixture models, typically focus on parameter estimation rather than defining and minimizing a notion of operator error. Work in [12] and [1] addresses the solution to Eq (1) in the context of clustering using a probabilistic theory of clustering for random labeled point sets and a definition of clustering error given by the expected number of “misclustered” points. This results in a *Bayes clusterer*, which minimizes error under the assumed probabilistic framework. An (optimal) Bayes clusterer is analogous to an (optimal) *Bayes* classifier, which minimizes classification error under the assumed feature-label distribution. Here, we characterize robust clustering using the framework and definitions of error in [12] and [1], and introduce definitions of robust clustering that parallel concepts from filtering. In particular, we present minimax, MCBR and IBR clusterers, and develop effective stochastic processes for robust clustering. We also evaluate performance under mixtures of Gaussians and demonstrate how the methodology can be implemented in practice with an example from granular imaging.

## Bayes clustering theory

In this section, we review Bayes clustering theory from [12] and [1]. A *random labeled point process* (RLPP) is characterized by a pair, (Ξ, Λ), where Ξ is a point process generating a point set $S\subset {\mathbb{R}}^{d}$ and Λ generates random labels on the points in *S*. In particular, let *η*(*S*) denote the number of points in *S*. The first component in this pair, Ξ, maps from a probability space to (N,N), where **N** is the family of finite sequences in ${\mathbb{R}}^{d}$ and N is the smallest *σ*-algebra on **N** such that for any Borel set *B* in ${\mathbb{R}}^{d}$ the mapping *S* ↦ *η*(*S* ∩ *B*) is measurable. A probability measure, *ν*, of Ξ is determined by the probabilities *ν*(*Y*) for Y∈N, or (via the Choquet-Matheron-Kendall theorem [13–16]), may be reduced to the system of probabilities *P*(Ξ ∩ *K* ≠ ∅) over all compact sets $K\subseteq {\mathbb{R}}^{d}$. Given a point set *S* ∈ **N**, a label function *ϕ*_*S*_: *S* → *L* = {1, 2, …, *l*} is a deterministic mapping that assigns each point **x** ∈ *S* to label *ϕ*_*S*_(**x**) ∈ *L*. The second component, Λ, is a random labeling, that is, Λ = {Φ_*S*_: *S* ∈ **N**}, where Φ_*S*_ is a random label function with probability mass *P*(Φ_*S*_ = *ϕ*_*S*_|*S*) on *L*^*S*^.

For any set *S*, and pair of label functions *ϕ*_*S*_ and *φ*_*S*_, define the *label mismatch error* between *ϕ*_*S*_ and *φ*_*S*_ to be the proportion of points where the label functions differ:

$$\begin{array}{c} \varepsilon \left(S,\phi_{S},\varphi_{S}\right)=\frac{1}{\eta (S)}\sum_{x\in S}\;I_{\phi_{S}\left(x\right)\neq \varphi_{S}\left(x\right)}, \end{array}$$ (5)

where *I*_*A*_ is an indicator function equal to 1 if *A* is true and 0 otherwise. Clustering involves identifying partitions of a point set rather than the actual labeling. A partition of *S* into *l* clusters has the form $P_{S}=\{S_{1},S_{2},\ldots ,S_{l}\}$ such that the *S*_*y*_ are disjoint and $S={⋃}_{y=1}^{l}S_{y}$. Every partition $P_{S}$ has associated with it a family, $G_{P_{S}}$, of label functions that induce the partition $P_{S}$. That is, $\varphi_{S}\in G_{P_{S}}$ if and only if $P_{S}=\{S_{1},S_{2},\ldots ,S_{l}\}$ where *S*_*y*_ = {**x** ∈ *S*: *φ*_*S*_(**x**) = *ℓ*_*y*_} and (*ℓ*_1_, …, *ℓ*_*l*_) is a permutation of *L*. For any point set *S*, label function *ϕ*_*S*_, and partition $P_{S}$, define the *cluster mismatch error* to be the minimum label mismatch error between *ϕ*_*S*_ and all label functions that induce $P_{S}$:

$$\begin{array}{c} \varepsilon \left(S,\phi_{S},P_{S}\right)=\underset{\varphi_{S}\in G_{P_{S}}}{\text{min}}\;\varepsilon \left(S,\phi_{S},\varphi_{S}\right). \end{array}$$ (6)

This is a simplified version of the original definition in [12]. Define the *partition error* of $P_{S}$ to be the mean cluster mismatch error over the distribution of label functions on *S*:

$$\begin{array}{cc} \varepsilon (S,P_{S}) & =E_{\Phi_{S}}\left[\varepsilon \left(S,\Phi_{S},P_{S}\right)|S\right]\\ & =E_{\Phi_{S}}[\underset{\varphi_{S}\in G_{P_{S}}}{\text{min}}\;\varepsilon \left(S,\Phi_{S},\varphi_{S}\right)|S]. \end{array}$$ (7)

In [1], it was shown that Eq (7) can be written in the form

$$\begin{array}{c} \varepsilon \left(S,P_{S}\right)=\sum_{Q_{S}\in K_{S}}\;c_{S}\left(P_{S},Q_{S}\right)P_{S}\left(Q_{S}\right), \end{array}$$ (8)

where $K_{S}$ is the set of all partitions of *S*,

$$\begin{array}{c} P_{S}\left(Q_{S}\right)=\sum_{\phi_{S}\in G_{Q_{S}}}\;P\left(\Phi_{S}=\phi_{S}|S\right) \end{array}$$ (9)

is the probability mass function on partitions $Q_{S}\in K_{S}$ of *S*, and we define the *natural partition cost function*,

$$\begin{array}{c} c_{S}\left(P_{S},Q_{S}\right)=\frac{1}{\eta (S)}\underset{\varphi_{S}\in G_{P_{S}},\phi_{S}\in G_{Q_{S}}}{\text{min}}\;\sum_{x\in S}\;I_{\phi_{S}\left(x\right)\neq \varphi_{S}\left(x\right)}. \end{array}$$ (10)

The partition error under the natural cost function is essentially the average number of misclustered points.

Taking Eq (8) as a generalized definition, other cost functions can be applied [17–20]. The natural cost function stands out in two respects. First, while these works define loss over label functions, we define cost directly over partitions, which is mathematically cleaner, and automatically treats the label switching problem in which multiple distinct label functions may produce the same partitions. Second, these works treat loss abstractly without connecting to a practical notion of clustering error, like the expected (minimum) number of mislabeled points. In contrast, we begin with a practical definition of clustering error, and prove that this error is equivalent to the average cost given in Eq (8) under the natural cost function.

A cluster operator *ζ* maps point sets to partitions. Define the *clustering error* of cluster operator *ζ* to be the mean partition error of *ζ*(Ξ) over the random point sets Ξ:

$$\varepsilon \left(\zeta \right)=E_{\Xi }\left[\varepsilon \left(\Xi ,\zeta \left(\Xi \right)\right)\right].$$

A *Bayes cluster operator* ζ* is a clusterer having minimal clustering error *ε*(ζ*), which is called the *Bayes clustering error*. Since *ε*(*S*, *ζ*(*S*)) depends on the clusterer *ζ* only at point set *S*, *ε*(*ζ*) is minimized by setting $\zeta^{*}(S)=P_{S}^{*}$ for all *S* ∈ **N**, where $P_{S}^{*}$ is a *Bayes partition* of *S*, defined to be a partition having minimal partition error, $\varepsilon (S,P_{S}^{*})$, called the *Bayes partition error*.

This formulation parallels classification theory, where an RLPP corresponds to a feature-label distribution, $\varepsilon (S,P_{S})$ corresponds to the probability that a given label is incorrect for a fixed point in the feature space, *ε*(*ζ*) corresponds to the overall classification error for an arbitrary classifier, ζ* corresponds to a Bayes classifier, and *ε*(ζ*) corresponds to the Bayes classification error.

To find the Bayes partition, we must evaluate the partition error for all partitions. We call partitions with non-zero probability *reference partitions*, and denote a set of *r* reference partitions by ${ℛ}_{S}=\{Q_{S}^{1},\ldots ,Q_{S}^{r}\}\subseteq K_{S}$. We call partitions that comprise the search space *candidate partitions*, and denote a set of *c* candidate partitions by $C_{S}=\{P_{S}^{1},\ldots ,P_{S}^{c}\}\subseteq K_{S}$. The set of candidate partitions is not required to contain all reference partitions, and may even contain non-reference partitions. The partition error of all candidate partitions is:

$$\begin{array}{cc} \left[\begin{array}{c} \varepsilon (S,P_{S}^{1})\\ \vdots \\ \varepsilon (S,P_{S}^{c}) \end{array}\right] & =[\begin{array}{ccc} c_{S}\left(Q_{S}^{1},P_{S}^{1}\right) & \cdots & c_{S}\left(Q_{S}^{r},P_{S}^{1}\right)\\ \vdots & \ddots & \vdots \\ c_{S}\left(Q_{S}^{1},P_{S}^{c}\right) & \cdots & c_{S}\left(Q_{S}^{r},P_{S}^{c}\right) \end{array}][\begin{array}{c} P_{S}\left(Q_{S}^{1}\right)\\ \vdots \\ P_{S}\left(Q_{S}^{r}\right) \end{array}]. \end{array}$$ (11)

If $C_{S}={ℛ}_{S}=K_{S}$, then we require a cost matrix of size $\left|K_{S}|\times |K_{S}\right|$, which can be prohibitively large for moderate *η*(*S*). To alleviate this, [1] provides both exact and approximate techniques to evaluate Eq (11) under the natural cost function with reduced complexity.

### Separable RLPPs

Up to this point, we have characterized RLPPs with a point process Ξ that generates point sets, *S*, followed by an *S*-conditioned labeling process Λ that generates label functions, *ϕ*_*S*_. Alternatively, it is often easier to characterize an RLPP as a process that draws a sample size *n*, a set of labels for *n* points, and a set of *n* points with distributions corresponding to the labels. For instance, one might think of points being drawn from *l* Gaussian distributions possessing random parameters. We say that an RLPP is *separable* if a label function *ϕ* is generated from an independent label generating process Φ with probability mass function *P*(Φ = *ϕ*) over the set of all label functions with domain {1, 2, …, *n*}, a random parameter vector *ρ* is independently drawn from a distribution *f*(*ρ*), and the *i*th point **x**_*i*_ in *S*, with corresponding label *y* = *ϕ*(*i*), is independently drawn from a conditional distribution *f*(**x**|*y*, *ρ*). From Bayes’ rule, the probability of label function *ϕ*_*S*_ ∈ *L*^*S*^ given *S* = {**x**_1_, …, **x**_*n*_} is

$$\begin{array}{c} P\left(\Phi_{S}=\phi_{S}|S\right)\propto f\left(S|\phi \right)P\left(\Phi =\phi \right), \end{array}$$ (12)

where *ϕ*(*i*) = *ϕ*_*S*_(**x**_*i*_),

$$\begin{array}{c} f\left(S|\phi \right)=\int (\prod_{y=1}^{l}\prod_{x\in S_{y}}\;f\left(x|y,\rho \right))f\left(\rho \right)d\rho , \end{array}$$ (13)

and *S*_*y*_ = {**x**_*i*_: *ϕ*(*i*) = *y*, *i* = 1, …, *n*} is the set of points in *S* assigned label *y*. A separable RLPP thus has three components: *P*(Φ = *ϕ*), *f*(*ρ*) and *f*(**x**|*y*, *ρ*), where *P*(Φ = *ϕ*) is a prior on labels, which is not dependent on *S*, and *P*(Φ_*S*_ = *ϕ*_*S*_|*S*) is a posterior probability on labels given a specific point set *S*, which is found using Eqs (12) and (13).

If *ρ* = [*ρ*_1_, …, *ρ*_*l*_], where the *ρ*_*y*_ are mutually independent parameter vectors and the label-*y*-conditional distribution depends on only *ρ*_*y*_, that is, if *f*(**x**|*y*, *ρ*) = *f*(**x**|*y*, *ρ*_*y*_) for *y* = 1, …, *l*, then,

$$\begin{array}{c} f\left(S|\phi \right)=\prod_{y=1}^{l}\int (\prod_{x\in S_{y}}\;f\left(x|y,\rho_{y}\right))f\left(\rho_{y}\right)d\rho_{y}. \end{array}$$ (14)

### Gaussian RLPPs

Expressions for label function probabilities have been solved under several models in [1]. Here, we review an important case in which clusters are Gaussian with random means and covariances. Specifically, consider a separable RLPP where, for each *y* ∈ {1, …, *l*}, *ρ*_*y*_ = [*μ*_*y*_, Σ_*y*_] and *f*(**x**|*y*, *ρ*_*y*_) is a Gaussian distribution with mean *μ*_*y*_ and covariance Σ_*y*_. Given a label function *ϕ*_*S*_, let *y* ∈ {1, …, *l*} be fixed, and let *n*_*y*_ be the number of points in *S* assigned label *y*. For *n*_*y*_ ≥ 2 it was shown in [1] that

$$\begin{array}{c} \prod_{x\in S_{y}}\;f\left(x|y,\rho_{y}\right)={\left(2\pi \right)}^{-\frac{dn_{y}}{2}}{\left|\Sigma_{y}\right|}^{-\frac{n_{y}}{2}}\text{exp}(-\frac{1}{2}\text{tr}(\Phi_{y}^{*}\Sigma_{y}^{-1})), \end{array}$$ (15)

where

$$\Phi_{y}^{*}=\left(n_{y}-1\right){\hat{\Sigma }}_{y}+n_{y}\left(\mu_{y}-{\hat{\mu }}_{y}\right){\left(\mu_{y}-{\hat{\mu }}_{y}\right)}^{T},$$

${\hat{\mu }}_{y}$ and ${\hat{\Sigma }}_{y}$ are the sample mean and covariance of points in *S*_*y*_, respectively, and where |⋅| denotes a determinant, *tr*(⋅) a trace and *T* a transpose. When *n*_*y*_ = 1, Eq (15) holds with $\Phi_{y}^{*}=\left(\mu_{y}-{\hat{\mu }}_{y}\right){\left(\mu_{y}-{\hat{\mu }}_{y}\right)}^{T}$, and when *n*_*y*_ = 0 the product over an empty set is 1.

Assume *f*(*ρ*_*y*_) = *f*(Σ_*y*_)*f*(*μ*_*y*_|Σ_*y*_), where *f*(*μ*_*y*_|Σ_*y*_) is a Gaussian distribution with mean **m**_*y*_ and covariance $\frac{1}{\nu_{y}}\Sigma_{y}$ with *ν*_*y*_ > 0, and *f*(Σ_*y*_) is an inverse-Wishart distribution with *κ*_*y*_ > *d* − 1 degrees of freedom and a positive-definite scale matrix Ψ_*y*_, i.e.,

$$f\left(\Sigma_{y}\right)=\frac{|\Psi_{y}{|}^{\frac{\kappa_{y}}{2}}{\left|\Sigma_{y}\right|}^{-\frac{\kappa_{y}+d+1}{2}}}{2^{\frac{\kappa_{y}d}{2}}\Gamma_{d}\left(\frac{\kappa_{y}}{2}\right)}\text{exp}\left(-\frac{1}{2}\text{tr}\left(\Psi_{y}\Sigma_{y}^{-1}\right)\right),$$

where Γ_*d*_ is the multivariate Gamma function. The expected mean is **m**_*y*_, the expected covariance matrix is $\frac{1}{\kappa_{y}-d-1}\Psi_{y}$ if *κ*_*y*_ > *d* + 1, and as *ν*_*y*_ and *κ*_*y*_ increase *f*(*ρ*_*y*_) becomes more “informative.” The probability of label function *ϕ*_*S*_ under this RLPP is found from Eqs (12) and (14) as

$$P(\Phi_{S}=\phi_{S}|S)\propto P\left(\Phi =\phi \right)\prod_{y=1}^{l}\frac{\Gamma_{d}\left(\frac{\kappa_{y}+n_{y}}{2}\right)}{|n_{y}+\nu_{y}{|}^{\frac{d}{2}}{\left|\Psi_{y}+\Psi_{y}^{*}\right|}^{\frac{\kappa_{y}+n_{y}}{2}}},$$ (16)

where

$$\begin{array}{c} \Psi_{y}^{*}=\left(n_{y}-1\right){\hat{\Sigma }}_{y}+\frac{\nu_{y}n_{y}}{\nu_{y}+n_{y}}\left({\hat{\mu }}_{y}-m_{y}\right){\left({\hat{\mu }}_{y}-m_{y}\right)}^{T} \end{array}$$ (17)

for *n*_*y*_ = 2, $\Psi_{y}^{*}=\frac{\nu_{y}}{\nu_{y}+1}\left({\hat{\mu }}_{y}-m_{y}\right){\left({\hat{\mu }}_{y}-m_{y}\right)}^{T}$ for *n*_*y*_ = 1, and $\Psi_{y}^{*}=0$ for *n*_*y*_ = 0. If *ν*_1_ = ⋯ = *ν*_*l*_, *κ*_1_ = ⋯ = *κ*_*l*_ and *P*(Φ = *ϕ*) is such that the size of each cluster is fixed and partitions with clusters of the specified sizes are equally likely, then for any *ϕ*_*S*_ inducing clusters of the correct sizes,

$$\begin{array}{c} P\left(\Phi_{S}=\phi_{S}|S\right)\propto \prod_{y=1}^{l}{\left|\Psi_{y}+\Psi_{y}^{*}\right|}^{-\frac{\kappa_{y}+n_{y}}{2}}. \end{array}$$ (18)

Similar derivations for the posterior on parameters under Gaussian mixture models can be found in [21], and similar derivations for the posterior on label functions under Gaussian mixture models can be found in [17].

## Robust clustering operators

Under a known RLPP (Ξ, Λ), optimization in the Bayes clusterer is over the set $\bar{C}$ of all clustering algorithms with respect to the clustering error,

$$\begin{array}{c} \zeta^{*}=\text{arg}\;\underset{\zeta \in \bar{C}}{\text{min}}\;\varepsilon \left(\zeta \right); \end{array}$$ (19)

however, in practice the RLPP is likely to be uncertain. In this section we present definitions for optimal Bayesian robust clustering and show that IBR clusterers solve an optimization problem of the same form as in Eq (19) under an effective process.

### Definitions of robust clustering

We present three robust clustering operators: minimax robust clustering, model-constrained Bayesian robust (MCBR) clustering, and intrinsically optimal Bayesian robust (IBR) clustering. Our main interest is in IBR clustering. The first two methods are provided to emphasize parallels between the new theory and existing robust operator theory from filtering and classification.

Consider a parameterized uncertainty class of RLPPs (Ξ_*θ*_, Λ_*θ*_), *θ* ∈ Θ, where Ξ_*θ*_ is a point process on (N,N), Λ_*θ*_ = {Φ_*θ*, *S*_: *S* ∈ **N**} is a random labeling on **N** consisting of a random label function Φ_*θ*, *S*_ for each *S*, and *ε*_*θ*_(*ζ*) is the error of cluster operator *ζ* for state *θ*.

A *minimax robust clusterer* *ζ*_MM_ is defined by Eq (2) with $C_{\Theta }$ being the set of state-specific Bayes clusterers and *ε*_*θ*_(*ζ*) in place of *γ*_*θ*_(*ψ*). An *MCBR cluster operator* *ζ*_MCBR_ is defined by Eq (3) with *ε*_*θ*_(*ζ*) in place of *γ*_*θ*_(*ψ*).

Our focus is on optimization over the full class $\bar{C}$ of cluster operators. This yields an *IBR cluster operator*,

$$\begin{array}{c} \zeta_{\text{IBR}}=\text{arg}\;\underset{\zeta \in \bar{C}}{\text{min}}\;E_{\theta }\left[\varepsilon_{\theta }\left(\zeta \right)\right]. \end{array}$$ (20)

In analogy to [2], where effective characteristics for IBR linear filtering were derived from effective random signal processes, here we show how IBR cluster operators can be found via effective random labeled point processes.

### Effective random labeled point processes

We begin with two definitions.

**Definition 1**. *An RLPP is solvable under clusterer class* C *if*

$$\zeta^{*}=\text{arg}\;\underset{\zeta \in C}{\text{min}}\;\varepsilon \left(\zeta \right)$$

*can be solved under this process*.

**Definition 2**. *Let* Θ *be an uncertainty class of RLPPs having prior π*(*θ*). *An RLPP* (Ξ_eff_, Λ_eff_) *is an effective RLPP under clusterer class* C *if for all* ζ∈C *both the expected clustering error E*_*θ*_[*ε*_*θ*_(*ζ*)] *under the uncertainty class of RLPPs and the clustering error ε*_eff_(*ζ*) *under* (Ξ_eff_, Λ_eff_) *exist and*

$$\begin{array}{c} E_{\theta }[\varepsilon_{\theta }\left(\zeta \right)]=\varepsilon_{\text{eff}}\left(\zeta \right). \end{array}$$ (21)

**Theorem 1**. *Let* Θ *parameterize an uncertainty class of RLPPs with prior π*(*θ*). *If there exists a solvable effective RLPP* (Ξ_eff_, Λ_eff_) *under clusterer class* C *with optimal clusterer* $\zeta_{eff}^{*}$, *then* $\zeta_{eff}^{*}=\text{arg}\;{\text{min}}_{\zeta \in C}E_{\theta }[\varepsilon_{\theta }(\zeta )]$. *If* $C=C_{\Theta }$, *then* $\zeta_{MCBR}^{*}=\zeta_{eff}^{*}$, *and if* $C=\bar{C}$, *then* $\zeta_{IBR}^{*}=\zeta_{eff}^{*}$.

*Proof*. The proof is immediate from the definition of an effective RLPP and Eq (19):

$$\text{arg}\;\underset{\zeta \in C}{\text{min}}\;E_{\theta }\left[\varepsilon_{\theta }\left(\zeta \right)\right]=\text{arg}\;\underset{\zeta \in C}{\text{min}}\;\varepsilon_{\text{eff}}\left(\zeta \right)=\zeta_{\text{eff}}^{*}.$$

The solutions for MCBR and IBR clustering follow from their definitions.

To find an MCBR or IBR clusterer, we first seek an effective RLPP. This effective RLPP is not required to be a member of the uncertainty class parameterized by *θ*, but must be solvable. If (Ξ_eff_, Λ_eff_) is an effective RLPP under clusterer class C, then it is an effective RLPP under any smaller clusterer class. Hence, an effective RLPP found for IBR clustering is also an effective RLPP for MCBR clustering. However, not only are IBR clusterers better performing than MCBR clusterers across the uncertainty class, they are typically much easier to find analytically. In particular, the IBR clusterer is directly solved by importing methods from Bayes clustering theory, i.e., one may solve Eq (19) by minimizing the partition error over all partitions of a point set *S* under the effective RLPP. The MCBR clusterer, on the other hand, is significantly hampered by computational overhead in finding $C_{\Theta }$ and actually evaluating the clustering error for each *ζ* ∈ *C*_Θ_. The next theorem addresses the existence of effective RLPPs.

**Theorem 2**. *Let* Θ *parameterize an uncertainty class* {(Ξ_*θ*_, Λ_*θ*_)}_*θ* ∈ Θ_ *of RLPPs with prior π*(*θ*). *There exists an RLPP*, (Ξ_eff_, Λ_eff_), *such that*

$$\begin{array}{c} E_{\theta }[E_{\Xi_{\theta },\Lambda_{\theta }}[g(\Xi_{\theta },\Phi_{\theta ,\Xi_{\theta }})|\theta ]]=E_{\Xi_{\text{eff}},\Lambda_{\text{eff}}}[g(\Xi_{\text{eff}},\Phi_{\text{eff},\Xi_{\text{eff}}})] \end{array}$$ (22)

*for any real-valued measurable function, g*.

*Proof*. Suppose that the parameter *θ* is a realization of a random vector, ϑ:(Ω,A,P)→(Θ,ℬ). Then {*ϑ*^−1^(*θ*): *θ* ∈ Θ} partitions the sample space, Ω. The point process Ξ_*θ*_ is thus a mapping

$$\Xi_{\theta }:\left(\vartheta^{-1}\left(\theta \right),A\cap \vartheta^{-1}\left(\theta \right),P_{\theta }\right)\to \left(N,N\right),$$

where *P*_*θ*_ is the conditional probability and we assume $\nu_{\theta }(Y)=P_{\theta }(\Xi_{\theta }^{-1}(Y))$ for all Y∈N is known. Write the random labeling as Λ_*θ*_ = {Φ_*θ*, *S*_: *S* ∈ **N**}, where Φ_*θ*,*S*_ has a probability mass function *P*(Φ_*θ*, *S*_ = *ϕ*_*S*_|*θ*, *S*) on *L*^*S*^. Given any real-valued measurable function *g* mapping from point set and label function pairs, let *X* = *g*(Ξ, Φ_Ξ_) be a random variable where (Ξ, Φ_Ξ_) is drawn from {(Ξ_*θ*_, Λ_*θ*_)}_*θ*∈Θ_ with prior *π*(*θ*), and note *E*_*θ*_[*E*[*X*|*θ*]] = *E*[*X*].

Let $\Xi_{eff}:(\Omega ,A,P)\to (N,N)$ be a mapping, where given a fixed *ω* ∈ Ω we have a corresponding fixed realization *θ* = *ϑ*(*ω*) and we define Ξ_eff_(*ω*) = Ξ_*θ*_(*ω*). Note that

$$\nu \left(Y\right)\equiv P\left(\Xi_{\text{eff}}^{-1}\left(Y\right)\right)=E_{\theta }\left[\nu_{\theta }\left(Y\right)\right]$$

and Ξ_eff_ is a random point process. Define Λ_eff_ = {Φ_eff,*S*_: *S* ∈ **N**}, where Φ_eff,*S*_ has a probability mass function

$$P\left(\Phi_{\text{eff},S}=\phi_{S}|S\right)=E_{\theta }\left[P\left(\Phi_{\theta ,S}=\phi_{S}|\theta ,S\right)\right]$$

for all *ϕ*_*S*_ ∈ *L*^*S*^. Thus, Λ_eff_ is a random labeling. Let $Z=g(\Xi_{eff},\Phi_{eff,\Xi_{eff}})$ be a random variable where $\left(\Xi_{eff},\Phi_{eff,\Xi_{eff}}\right)$ is drawn from the RLPP we have constructed, (Ξ_eff_, Λ_eff_), and note *E*[*X*] = *E*[*Z*].

Theorem 2 applies for any function *g*(*S*, *ϕ*_*S*_), including the cluster mismatch error *g*(*S*, *ϕ*_*S*_) = *ε*(*S*, *ϕ*_*S*_, *ζ*(*S*)), for any clusterer $\zeta \in \bar{C}$. Thus, Eq (22) implies

$$\begin{array}{cc} E_{\theta }[\varepsilon_{\theta }\left(\zeta \right)] & =E_{\theta }[E_{\Xi_{\theta },\Lambda_{\theta }}[\varepsilon (\Xi_{\theta },\Phi_{\theta ,\Xi_{\theta }},\zeta \left(\Xi_{\theta }\right))|\theta ]]\\ & =E_{\Xi_{\text{eff}},\Lambda_{\text{eff}}}[\varepsilon (\Xi_{\text{eff}},\Phi_{\text{eff},\Xi_{\text{eff}}},\zeta \left(\Xi_{\text{eff}}\right))]=\varepsilon_{\text{eff}}\left(\zeta \right). \end{array}$$

Hence, (Ξ_eff_, Λ_eff_) is an effective RLPP on $\bar{C}$, covering MCBR and IBR clusterers.

The following corollary shows that for separable RLPPs, the effective RLPP is also separable and aggregates uncertainty within and between models.

**Corollary 1**. *Let each RLPP in the uncertainty class be parameterized by ρ with prior density f*(*ρ*|*θ*), *let* Φ *be an independent labeling process with a probability mass P*(Φ = *ϕ*) *that depends on neither θ nor ρ, and denote the conditional distribution of points by f*(**x**|*y*, *ρ*, *θ*). *Then the effective RLPP is separable with parameter* [*θ*, *ρ*], *prior f*(*θ*, *ρ*), *an independent labeling process with probability mass P*(Φ = *ϕ*), *and conditional distributions f*(**x**|*y*, *ρ*, *θ*).

*Proof*. Let the number of points, *n*, and the label function *ϕ*: {1, …, *n*}→*L*^*n*^ be fixed. For a fixed *θ*, the effective random point process Ξ_eff_(*ω*) is set equal to Ξ_*θ*_(*ω*). Equivalently, a realization of *S* = {**x**_1_, …, **x**_*n*_} under the effective RLPP is governed by the distribution

$$f\left(S|\phi \right)=\int (\int (\prod_{i=1}^{n}f\left(x_{i}|\phi \left(i\right),\rho ,\theta \right))f\left(\rho |\theta \right)d\rho )\pi \left(\theta \right)d\theta .$$

This is equivalent to a separable random point process with parameter [*θ*, *ρ*], prior *f*(*θ*, *ρ*) = *π*(*θ*)*f*(*ρ*|*θ*) and conditional distributions *f*(**x**|*y*, *ρ*, *θ*). Since the labeling process is independent, the full effective RLPP is the separable RLPP given in the statement of the corollary.

**Fig 1:**
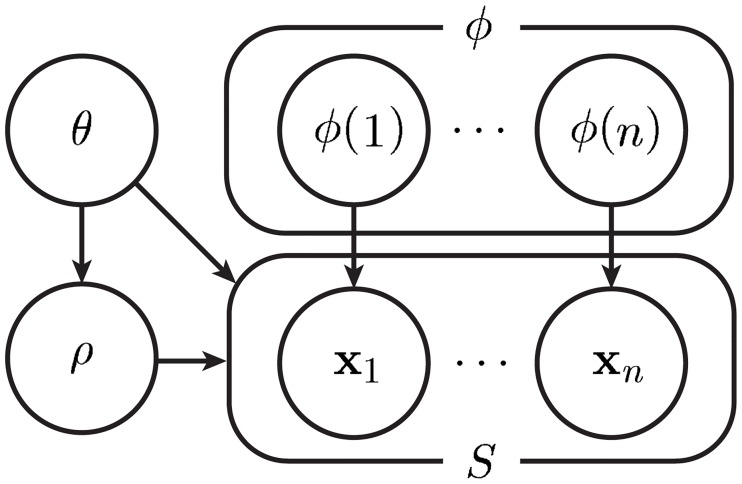
A graphical model of the uncertainty class of RLPPs assumed in Corollary 1. The parameter *θ* is governed by a prior distribution *π*(*θ*) and indexes each RLPP in the uncertainty class. The number of points, *n*, may be generated from an independent process, or considered fixed. For fixed *n*, the label function, *ϕ*, is generated according to the probability mass function *P*(Φ = *ϕ*). Given *θ*, *ρ* is generated from the density *f*(*ρ*|*θ*), and each point in the point set *S* = {**x**_1_, …, **x**_*n*_} is drawn from the density *f*(**x**|*y*, *ρ*, *θ*), where the corresponding label for point **x**_*i*_ is *y* = *ϕ*(*i*).

A graphical model of the uncertainty class of RLPPs assumed in Corollary 1 is provided in Fig 1. A general description of how the IBR clusterer may be found follows.

1. We assume an uncertainty class of RLPPs of the form stated in Corollary 1 and illustrated in Fig 1. In particular, we require the sample size, *n*, prior *π*(*θ*), label process probability mass function *P*(Φ = *ϕ*), parameter prior *f*(*ρ*|*θ*) and conditional density *f*(**x**|*y*, *ρ*, *θ*). These components characterize our prior knowledge about the point set generating process, and ideally are constructed and validated using available scientific knowledge about the problem at hand.
2. By Corollary 1, the effective RLPP is found by merging uncertainty in the state (across RLPPs) and parameters (within RLPPs). In particular, the effective RLPP is characterized by the sample size *n*, label process probability mass function *P*(Φ = *ϕ*), parameter prior *f*(*θ*, *ρ*) and density *f*(**x**|*y*, *ρ*, *θ*).
3. By Theorem 1, the IBR clusterer is the Bayes (optimal) clusterer under the effective RLPP. Given point set *S*, the IBR clusterer outputs the partition $P_{S}$ corresponding to the minimal error $\varepsilon (S,P_{S})$ in Eq (11). The natural cost function *c*_*S*_ is a constant function given by Eq (10), the partition probabilities are given by Eq (9), and the label function probabilities *P*(Φ_*S*_ = *ϕ*_*S*_|*S*) under the effective (separable) RLPP are given by Eq (12) with the likelihood function in Eq (13) using *f*(*θ*, *ρ*) in place of *f*(*ρ*) and *f*(**x**|*y*, *ρ*, *θ*) in place of *f*(**x**|*y*, *ρ*). An algorithmic description of the IBR clusterer under separable RLPPs is provided in Algorithm 1. Algorithm 1 is equivalent to the Bayes clusterer under the effective RLPP, and several optimal and suboptimal methods to improve upon this Bayes clustering algorithm are provided in [1].

In practice, the primary issues are: (a) deriving an analytical form for the label function probability, *P*(Φ_*S*_ = *ϕ*_*S*_|*S*), (b) evaluating the natural cost, *c*_*S*_, for all pairs of partitions, and (c) evaluating partition errors, $\varepsilon (S,P_{S})$, for all partitions. Note that *P*(Φ_*S*_ = *ϕ*_*S*_|*S*) is available for Gaussian separable RLPPs in Eq (16). Issues (b) and (c) may also be alleviated using optimal and suboptimal algorithms, as discussed in [1].

**Algorithm 1** IBR Clustering for Separable RLPPs

**Require:** Data set, *S*

**Require:** Maximum number of clusters, *l*

**Require:** Label generating process, *P*(Φ = *ϕ*)

**Require:** Effective parameter generation process, *f*(*θ*, *ρ*)

**Require:** Effective data generation process, *f*(**x**|*y*, *ρ*, *θ*)

 *n* ← number of points in *S*

 $K_{S}\leftarrow$ set of all possible partitions on *n* points with up to *l* clusters

 *r* ← number of partitions in $K_{S}$ (number of reference partitions)

 *c* ← number of partitions in $K_{S}$ (number of candidate partitions)

 *normalize* ← 0

 **for** *i* = 1 to *r* **do**

  $Q_{S}\leftarrow K_{S}(i)$

  **for all** label vectors *ϕ* that induce partition $Q_{S}$ **do**

   *f*(*S*|*ϕ*) ← likelihood from Eq (13) using *f*(*θ*, *ρ*) and *f*(**x**|*y*, *ρ*, *θ*)

   *a*(*ϕ*) ← *f*(*S*|*ϕ*)*P*(Φ = *ϕ*) (unnormalized label function prob. from Eq (12))

   *normalize* ← *normalize* + *a*(*ϕ*)

  **end for**

 **end for**

 **for** *i* = 1 to *r* **do**

  $Q_{S}\leftarrow K_{S}(i)$

  *sum* ← 0

  **for all** label vectors *ϕ*_*S*_ that induce partition $Q_{S}$ **do**

   *P*(Φ_*S*_ = *ϕ*_*S*_|*S*) ←*a*(*ϕ*)/*normalize* (normalized label function probability)

   *sum* ← *sum* + *P*(Φ_*S*_ = *ϕ*_*S*_|*S*)

  **end for**

  *P*_*S*_(*i*) ← *sum* (partition probability from Eq (9))

 **end for**

 **for** *j* = 1 to *c* **do**

  $P_{S}\leftarrow K_{S}(j)$

  **for** *i* = 1 to *r* **do**

   $Q_{S}\leftarrow K_{S}(i)$

   *c*_*S*_(*j*, *i*) ← natural cost between $P_{S}$ and $Q_{S}$ from Eq (10)

  **end for**

  $\varepsilon (S,j)\leftarrow \sum_{i=1}^{r}c_{S}(j,i)P_{S}(i)$ (clustering error from Eq (8))

 **end for**

 j* ← min_*j* = 1, …, *c*_ *ε*(*S*, *j*)

 $P_{S}^{*}\leftarrow K_{S}(j^{*})$ (output the IBR partition)

## Robust clustering under Gaussian RLPPs

Consider synthetic Gaussian data with *l* = 2 clusters in *d* = 1, 2, 10, 100 and 1, 000 dimensions. The state of nature is composed of the cluster covariances, and for a given state of nature the point process generates equal sized Gaussian clusters with random means and the corresponding covariances. Formally, we parameterize the uncertainty class of RLPPs with *θ* = [*θ*_1_, *θ*_2_], where *θ*_*y*_ = Σ_*y*_, and each Σ_*y*_ is drawn independently from an inverse-Wishart distribution with *κ*_*y*_ degrees of freedom and scale matrix Ψ_*y*_. The RLPP in the uncertainty class corresponding to *θ*, (Ξ_*θ*_, Λ_*θ*_), is a separable RLPP with parameter *ρ*_*y*_ = *μ*_*y*_, Gaussian prior *f*(*ρ*_*y*_|*θ*_*y*_) with mean **m**_*y*_ and covariance Σ_*y*_/*ν*_*y*_, and Gaussian conditional distributions *f*(**x**|*y*, *ρ*_*y*_, *θ*_*y*_) with mean *μ*_*y*_ and covariance Σ_*y*_. We set *κ*_1_ = *κ*_2_ = *d* + 2, Ψ_1_ = Ψ_2_ to be *d* × *d* identity matrices, *ν*_1_ = *ν*_2_ = 1, and **m**_1_ = **m**_2_ to be all-zero vectors. The number of points, *n* = *n*_1_+*n*_2_, is set to 10 or 100 with *n*_1_ = *n*_2_, and the labels are permuted. Thus, the true distribution on label functions, *P*(Φ = *ϕ*), has a support on the set of label functions that assign the correct number of points to each cluster, and is uniform on its support.

For each combination of *d* and *n*, we generate 1, 000 states of nature, *θ*, and one point set per state of nature from the corresponding separable RLPP (Ξ_*θ*_, Λ_*θ*_). For each point set, we run several classical clustering algorithms: fuzzy *c*-means (FCM), *k*-means (KM), hierarchical clustering with single linkage (H-S), hierarchical clustering with average linkage (H-A), hierarchical clustering with complete linkage (H-C), and a clusterer that produces a random partition with equal sized clusters for reference (Random). More details about these algorithms may be found in [22]. In addition, we cluster using expectation maximization for Gaussian mixture models (EM), and a method that minimizes a lower bound on the posterior expected variation of information under an estimated posterior similarity matrix generated from samples of a Gibbs sampler for Gaussian mixture models (MCMC) [23]. EM is run using the mclust package in R with default settings [24–26]. The Gibbs sampler is implemented using the bayesm package in R with 18, 000 samples generated after a burn-in period of 2, 000 samples, and otherwise default settings [27]. The posterior similarity matrix is estimated using the mcclust package in R [28], and minimization with respect to variation of information is implemented with the mcclust.ext package in R [29]. We also implement EM informed with the “correct” hyperparameters, *κ*_*y*_, Ψ_*y*_, *ν*_*y*_ and **m**_*y*_ (EM-I) and MCMC informed with the “correct” hyperparameters (MCMC-I).

To find the IBR clusterer, the effective RLPP, (Ξ, Λ), is constructed using Corollary 1, which states that the effective RLPP merges uncertainty in the state *θ* with uncertainty in the parameter *ρ*. In this case, the effective RLPP is precisely the separable RLPP presented in the “Gaussian RLPPs” section, which accounts for both random means in *ρ* and random covariances in *θ*. The effective RLPP is solvable (at least for small point sets) using the Bayes clusterer presented in [1]. By Theorem 1, the IBR clusterer is equivalent to the Bayes clusterer under the effective RLPP. Thus, the IBR clusterer can be found when *n* = 10 by evaluating Eq (8) for all partitions using Eqs (9) and (18), and choosing the minimizing partition. When *n* = 100, we approximate the IBR clusterer (IBR-A) using a sub-optimal algorithm, Suboptimal Pseed Fast, presented in [1], which finds the maximum probability partition for a random subset of 10 points, generalizes these clusters to the full point set using a QDA classifier (in this case the threshold is selected such that *n*_1_ = *n*_2_), iteratively searches for the highest probability partition on the full point set by considering all partitions with at most two points clustered differently from the best partition found so far, and finally chooses the highest probability partition resulting from 10 repetitions with different initial subsets of points. MCBR and minimax robust clusterers are not found, since they are computationally infeasible. Furthermore, having found an IBR clusterer one would certainly not use an MCBR clusterer and very likely not use a minimax robust clusterer.

**Fig 2:**
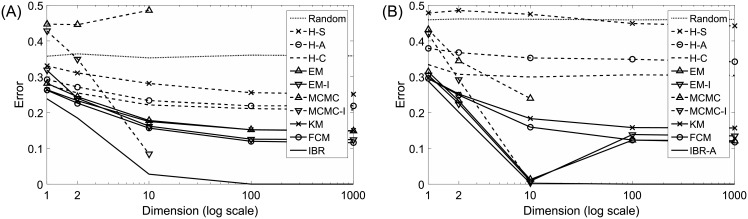
Average cluster mismatch error for Gaussian RLPPs. (A) *n* = 10. (B) *n* = 100.

For each point set and each algorithm, we find the cluster mismatch error between the true partition and the algorithm output using Eq (6). For each combination of *d* and *n* and each algorithm, we approximate the average clustering error, *E*_*θ*_[*ε*_*θ*_(*ζ*)], under the natural cost function using the average cluster mismatch error across all 1, 000 point sets. Fig 2A presents a graph of these errors with respect to *d* for *n* = 10, and similarly Fig 2B presents performance for *n* = 100. In part A the IBR clusterer is optimal. Although the IBR clusterer cannot be found in part B for *n* = 100, it is expected that its performance curve here should be slightly lower than its performance curve in part A for *n* = 10 [1]. Note the approximate IBR clusterer in part B is slightly higher than the IBR clusterer in part A for 1 and 2 dimensions, slightly lower for 10 dimensions, and they both have nearly zero error for 100 and 1, 000 dimensions (in fact, they are the only algorithms with error rates below 10% in these high-dimension cases). This suggests that the IBR clusterer is very close to optimal, especially in easy problems where the error rate is very small.

When the number of points is large (*n* = 100) and the number of dimensions is smaller than the number of points, the performances of EM and EM-I are very close to the approximate IBR clusterer. However, when the number of points is small, or the number of dimensions is larger than the number of points, these algorithms tend to be similar to FCM and KM. This is most likely because mclust tests several different modeling assumptions regarding the covariances, and uses the Bayesian information criterion (BIC) to select a final output partition. When *n* is small relative to *d*, the full covariances of the Gaussian mixtures cannot be estimated well, so there is a tendency to select simpler models that assume covariances are equal and circular, which is essentially the same assumption made by FCM and KM. MCMC by default uses a particular normal-inverse-Wishart prior with hyperparameters that do not match the “correct” hyperparameters. The fact that MCMC-I performs much better than MCMC suggests that this method may be quite sensitive to the priors, especially when the sample size is small. Finally, note that MCMC and MCMC-I are not shown for *d* = 100 because they tend to be unstable in this case. For example, when *n* = 100 and *d* = 100 MCMC outputs all points in the same cluster for 913 out of 1, 000 iterations, MCMC-I outputs a label vector with the alternating pattern “1, 2, 1, 2, …” (i.e., it always assigns odd and even indexed points to separate clusters) for 849 out of 1, 000 iterations, and MCMC-I outputs this alternating pattern with at most three of the *n* = 100 points being exceptions for all of the remaining iterations. MCMC and MCMC-I are not shown for *d* = 1, 000 because the code crashes in this case.

Sample size does not influence the performance of IBR, IBR-A, KM or FCM very much, nor the performance of EM or EM-I under large dimensions. When dimensionality is small, EM and EM-I improve as we increase sample size, to the point where they approach the performance of the (nearly optimal) approximate IBR clusterer. In cases where MCMC and MCMC-I are working, they also improve as sample size increases, though to a lesser extent. One reason that EM and MCMC based methods may not perform particularly well under small samples is that they attempt to estimate the underlying Gaussian densities behind the clusters, and estimating an arbitrary covariance matrix becomes problematic as dimensionality increases. In contrast, the IBR clusterer does not attempt to estimate the densities, but rather directly focuses on an easier problem: finding the best partition of the points. The performance of hierarchical methods all degrade as sample size increases, with H-S actually performing worse than random clustering in some cases. Hierarchical methods are sensitive to outliers and certain artifacts in the data that may be more likely to occur under large samples.

When the IBR clusterer assumes *n*_1_ = *n*_2_, there are *r* = *C*(*n*, *n*_1_)/2 reference partitions and *c* = 2^*n*−1^ candidate partitions. For *n* = 10, *r* = 126 and *c* = 512 and the cost matrix in (11) contains 126 × 512 = 64, 512 elements. Although we use methods described in [1] to find the Bayes clusterer efficiently without computing the whole cost matrix whenever possible (typically these tricks are most effective when the error rate is low), the size of the cost matrix increases very rapidly as *n* increases. The IBR clusterer is currently infeasible to compute for more than about *n* = 20, which corresponds to *r* = 92, 378 reference and *c* = 524, 288 candidate partitions. Run times for algorithms run in the *n* = 100 case are shown in Table 1. Note we initialize IBR-A using the computationally intensive maximum probability partition for the subset of 10 points to improve error rates; when initializing with FCM run time improves at the expense of performance. An extensive analysis of the runtime and memory requirements for IBR and the FCM-based variant of IBR-A are provided in [1].

**Table 1:** Average run time (in ms) over 1, 000 iterations in the Gaussian example with *n* = 100.

*d*	IBR-A	FCM	KM	MCMC	MCMC-I	EM	EM-I	H-C	H-A	H-S	Random
1	4, 266	1.2	3.4	12, 436	12, 047	5.3	4.6	1.1	1.1	1.3	0.5
2	17, 077	1.2	3.2	11, 461	11, 256	21.7	15.6	1.1	1.1	1.4	0.3
10	15, 992	1.5	3.3	12, 416	11, 965	405.3	18.0	1.1	1.1	1.4	0.3
100	172, 104	5.8	4.0	142, 888	147, 804	18.4	20.2	1.3	1.2	1.6	0.3
1,000	241, 375	26.9	10.3	–	–	171.2	191.2	2.9	2.8	3.2	0.4

## Robust clustering in granular imaging

While digital photography may now dominate over chemical photography, silver-based imaging remains important and is currently growing in use. Research remains active. Crystal shape is of particular importance. For many years granulometric analysis has been important in particle and texture analysis. In particular, morphological granulometries can generate image features relating to the size, shape, and concentration of particles. We present an application of robust clustering for images of silver-halide photographic T-grain crystals with respect to grain proportions using granulometric features.

### Morphological granulometries

A basic model for silver-halide emulsions includes grains that are equilateral triangles, hexagons formed by removing triangle corners, rods (rectangles), and ill-formed blobs. To simplify calculations, we focus on a binary image model using only triangles and rods. In film grade emulsions grains overlap, but for laboratory analysis diluted emulsions with negligible overlapping can be produced, thus we also focus on images with non-overlapping grains.

Morphological granulometries are particularly well-suited for modeling and processing binary images consisting of grains of different sizes and shapes. The most commonly employed granulometry is a family of parameterized morphological openings: for a convex, compact *structuring element* (set) *B*, a *granulometry* {Ψ_*t*_} is defined by Ψ_*t*_(*I*) = *I* ∘ *tB* for *t* > 0 and Ψ_0_(*I*) = *I*, where *I* ∘ *tB* = ∪{*tB* + *x*: *tB* + *x* ⊂ *I*} is the opening of image (set) *I* by *tB* (more general granulometries exist [15]). If Ω_*I*_(*t*) is the area of Ψ_*t*_(*I*), then Ω_*I*_(*t*) is a decreasing function of *t*, known as a *size distribution*. A normalized size distribution is defined by Φ_*I*_(*t*) = 1 − Ω_*I*_(*t*)/Ω_*I*_(0). If *I* is compact and *B* consists of more than a single point, then Φ_*I*_(*t*) increases from 0 to 1 and is continuous from the left. Thus, it defines a probability distribution function called the *pattern spectrum* of *I* (relative to *B*). Moments of Φ_*I*_(*t*) are used for image classification and segmentation [30]. Φ_*I*_(*t*) is a random function and its non-central moments (called *granulometric moments*) are random variables.

In this work, we use granulometric moments as features for clustering. Given a set *I*, we extract as features the first *q* granulometric moments of *I* generated by granulometries arising from *p* structuring elements *B*_1_, *B*_2_, …, *B*_*p*_, where we denote the *k*th granulometric moment corresponding to *B*_*j*_ by *μ*^(*k*)^(*I*, *B*_*j*_) for *j* = 1, 2, …, *p* and *k* = 1, 2, …, *q*. Consider a random set *I* of the form

$$\begin{array}{c} I=\overset{m}{\underset{i=1}{\cup }}\overset{N_{i}}{\underset{j=1}{\cup }}(r_{ij}A_{i}+x_{ij}), \end{array}$$ (23)

where *A*_1_, *A*_2_, …, *A*_*m*_ are compact sets called *primitives*, *r*_*ij*_ and *x*_*ij*_ specify the radius (grain size) and center of the *j*th grain of primitive type *i*, respectively, and all *N* = *N*_1_ + … + *N*_*m*_ grains are mutually disjoint.

In the silver halide application, we assume preprocessed images are well modeled by Eq (23), where *m* = 2, *A*_1_ is an equilateral triangle with horizontal base, and *A*_2_ is a vertical rod with height 5 times its base. Without loss of generality, we assume both primitives have unit area, i.e., *ν*[*A*_1_] = *ν*[*A*_2_] = 1, and we denote the grain proportions by *b*_1_ and *b*_2_. We further assume the *r*_*ij*_ are independent with the $r_{i1},\ldots ,r_{iN_{i}}$ identically distributed, where the *grain sizing distribution* for primitive *i* has the property $E[r_{ij}^{k}]=\gamma_{ik}\beta^{k}$ for all *k* > 0 and *γ*_*ik*_ and *β* are positive constants. If *r*_*ij*_∼ gamma(*α*_*i*_, *β*), *β* being the scale parameter for both primitives, then this property holds with *γ*_*ik*_ = Γ(*α*_*i*_ + *k*)/Γ(*α*_*i*_).

For the morphological opening, we use *p* = 2 structuring elements, where *B*_1_ and *B*_2_ are, respectively, vertical and horizontal linear structuring elements. The first *q* = 2 granulometric moments for *B*_1_ and *B*_2_ are

$$z={\left[\begin{array}{c} \mu^{\left(1\right)}\left(I,B_{1}\right)\;\mu^{\left(1\right)}\left(I,B_{2}\right)\;\mu^{\left(2\right)}\left(I,B_{1}\right)\;\mu^{\left(2\right)}\left(I,B_{2}\right) \end{array}\right]}^{T}.$$

Given the constants *μ*^(*k*)^(*A*_*i*_, *B*_*j*_) and the radii *r*_*ij*_ of all grains, the exact moments in **z** under the granulometric model may be found analytically (see Theorem 1 in S1 Appendix). In particular, **z** = *M***x**, where

$$M=[\begin{array}{cccc} \mu^{\left(1\right)}\left(A_{1},B_{1}\right) & \mu^{\left(1\right)}\left(A_{2},B_{1}\right) & 0 & 0\\ \mu^{\left(1\right)}\left(A_{1},B_{2}\right) & \mu^{\left(1\right)}\left(A_{2},B_{2}\right) & 0 & 0\\ 0 & 0 & \mu^{\left(2\right)}\left(A_{1},B_{1}\right) & \mu^{\left(2\right)}\left(A_{2},B_{1}\right)\\ 0 & 0 & \mu^{\left(2\right)}\left(A_{1},B_{2}\right) & \mu^{\left(2\right)}\left(A_{2},B_{2}\right) \end{array}],$$

and **x** = [*x*_11_, *x*_21_, *x*_12_, *x*_22_]^*T*^, where

$$\begin{array}{c} x_{ik}=\frac{\sum_{j=1}^{N_{i}}r_{ij}^{k+2}}{\sum_{j=1}^{N_{1}}r_{1j}^{2}+\sum_{j=1}^{N_{2}}r_{2j}^{2}}. \end{array}$$ (24)

In general, the constants *μ*^(*k*)^(*A*_*i*_, *B*_*j*_) under convex grains can be found using theory from [31]. It can be shown that for triangle *A*_1_ and vertical structuring element *B*_1_ that *μ*^(1)^(*A*_1_, *B*_1_) = 2 ⋅ 3^−3/4^ and *μ*^(2)^(*A*_1_, *B*_1_) = 2^−1^3^1/2^. Similarly, for other combinations of primitives and structuring elements, *μ*^(1)^(*A*_1_, *B*_2_) = 4 ⋅ 3^−5/4^, *μ*^(2)^(*A*_1_, *B*_2_) = 2 ⋅ 3^−1/2^, *μ*^(1)^(*A*_2_, *B*_1_) = 5^1/2^, *μ*^(2)^(*A*_2_, *B*_1_) = 5, *μ*^(1)^(*A*_2_, *B*_2_) = 5^−1/2^ and *μ*^(2)^(*A*_2_, *B*_2_) = 5^−1^.

In the current application, we cluster on the features **x** = *M*^−1^**z**. Theorem 3 in S1 Appendix. guarantees asymptotic joint normality and provides analytic expressions for the asymptotic mean and covariance of granulometric moments under multiple primitives and multiple structuring elements. In particular, given the grain proportions *b*_1_ and *b*_2_, and the grain sizing parameters *β* and *γ*_*ik*_ for *i* = 1, 2 and *k* = 2, 3, 4, **x** has asymptotic mean

$$\begin{array}{c} \frac{1}{b_{1}\gamma_{12}+b_{2}\gamma_{22}}{\left[\begin{array}{cccc} b_{1}\gamma_{13}\beta & b_{2}\gamma_{23}\beta & b_{1}\gamma_{14}\beta^{2} & b_{2}\gamma_{24}\beta^{2} \end{array}\right]}^{T} \end{array}$$ (25)

and covariance matrix

$$\begin{array}{c} \frac{1}{N{\left(b_{1}\gamma_{12}+b_{2}\gamma_{22}\right)}^{4}}[\begin{array}{cc} A_{11}\beta^{2} & A_{12}\beta^{3}\\ A_{21}\beta^{3} & A_{22}\beta^{4} \end{array}], \end{array}$$ (26)

where the *A*_*ij*_ are 2 × 2 matrices that depend on only the *b*_*i*_ and *γ*_*ik*_, and are provided in S1 Appendix.

### Robust clustering

Suppose we are given a collection of *n* binary images of mixtures of silver-halide photographic T-grain crystals, where each image belongs to one of two groups, indexed by *y* = 1, 2. Images in class 1 and 2 have different proportions of triangles, *b*_1_, and different sizing parameters, thereby providing different photographic properties. Our objective is to cluster the images into the two groups (our concern is partitioning, not labeling) based on feature vectors **x** = *M*^−1^**z** obtained from moments of morphological openings **z**.

Given the grain sizing distributions and a prior *f*(*ρ*), the asymptotic joint normality of **x** motivates a separable RLPP model where, given *y* and *ρ*, *f*(**x**|*y*, *ρ*) is a Gaussian distribution with mean and covariance given by Eqs (25) and (26), respectively. We substitute *ρ* and 1−*ρ* in place of *b*_1_ and *b*_2_ under class 1, and vice-versa under class 2. For simplicity, we assume *P*(Φ = *ϕ*) is uniform with support such that the number of images in each class is known.

The grain sizing distribution in a binarized image typically depends on the image thresholding method and other factors, and thus is unknown. To account for this, we model an uncertainty class of RLPPs parameterized by *θ*, where the grain sizes are assumed to be gamma(*α*_*iy*_, *β*_*y*_) distributed, the *α*_*iy*_ parameters are fixed and known, the *β*_*y*_ depend deterministically on *θ*, and we assume *θ* and *ρ* are mutually independent with known prior *π*(*θ*). From Eqs (12) and (13), the IBR clusterer reduces to finding the following label function probabilities under the effective RLPP:

$$\begin{array}{cc} & P\left(\Phi_{S}=\phi_{S}|S\right)\propto P\left(\Phi =\phi \right)\times \\ & \;\int_{0}^{\infty }\int_{0}^{1}f\left(S_{1}|1,\rho ,\theta \right)f\left(S_{2}|2,\rho ,\theta \right)f\left(\rho \right)\pi \left(\theta \right)d\rho d\theta , \end{array}$$ (27)

where

$$f\left(S_{y}|y,\rho ,\theta \right)=\prod_{x\in S_{y}}\;f\left(x|y,\rho ,\theta \right).$$

Since we assume a Gaussian model, *f*(*S*_*y*_|*y*, *ρ*, *θ*) is precisely the likelihood function in Eq (15). To make Eq (27) tractable, we assume discrete priors on *ρ* and *θ* so that the integrals can be written as sums.

### Experimental results

**Fig 3:**
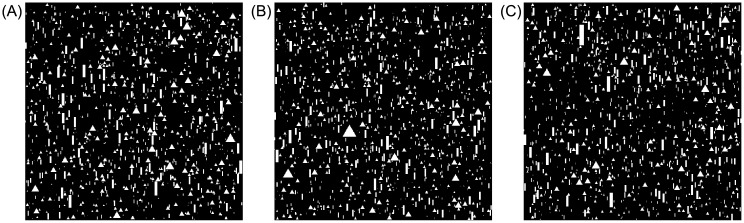
Examples of image realizations generated by the T-grain crystal model. Each image contains 1, 000 grains. The sizing distribution of the grains are gamma(*α* = 1.95, *β* = 2) for the triangles and gamma(*α* = 1.97, *β* = 2) for the rods. The size of each image is 550 × 550 pixels. (A) Proportions of 0.45 triangles and 0.55 rods. (B) 0.5 triangles and 0.5 rods. (C) 0.55 triangles and 0.45 rods.

The image generation model is based on the parameterized RLPP model described above. For a given set of images under a given RLPP with parameter *θ*, which determines the sizing distribution, we generate *n* = *n*_1_ + *n*_2_ binary images, where *n*_1_ and *n*_2_ denote the fixed number of images from class 1 and class 2, respectively. Each image contains 1, 000 non-overlapping and vertically aligned grains (triangles and rods), and is 550 × 550 pixels. The prior *f*(*ρ*) on the proportion of triangles for class 1 is uniform over 500 values from 0.45 to 0.55, and we assume the proportion of triangles for class 2 is 1 − *ρ*. Fig 3 shows three example realizations of images with gamma(*α* = 1.95, *β* = 2) sizing distributions for the triangles and gamma(*α* = 1.97, *β* = 2) for the rods. Parts A, B, and C contain triangle proportions 0.45, 0.5, and 0.55, respectively.

**Fig 4:**
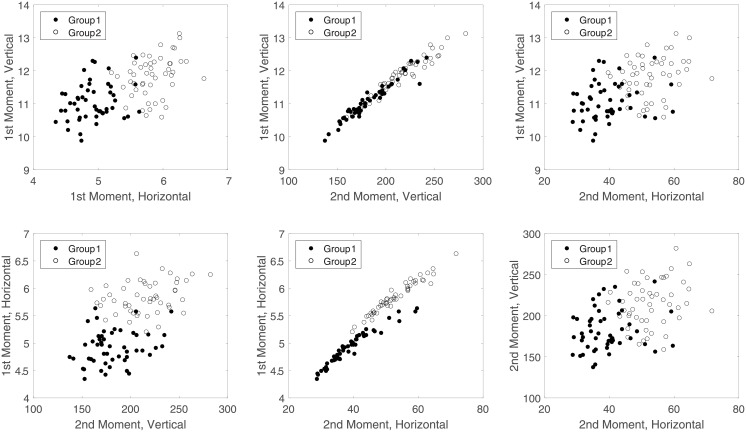
Scatter plots of all pairs of features extracted from 100 images.

The prior *π*(*θ*) is uniform over 10 values from 1.75 to 2. We assume gamma(*α*_*iy*_, *β*_*y*_) sizing distributions for primitive *i* under class *y*, where *β*_1_ = *θ*, *β*_2_ = 3.75 − *θ*. For triangles, *α*_1*y*_ = 1.95 and 1.97 for class 1 and class 2, respectively, and for rods, *α*_2*y*_ = 1.97 and 1.95 for class 1 and class 2, respectively. We generate 500 sets of images for each state, for a total of 5, 000 sets of images. For each image, openings are found, followed by granulometric moments **z** from the openings, and finally a feature vector **x** = *M*^−1^**z**. Fig 4 provides example scatter plots of all pairs of features extracted from 100 images. These images correspond to *θ* = 1.75 and the 10 smallest values of *ρ* (between 0.45 and 0.452), with 5 images selected from each value of *ρ* and each group.

For each set of images, we run FCM, KM, H-S, H-A, H-C, EM, MCMC and Random. Note EM-I and MCMC-I, which use normal-inverse-Wishart priors on the mean and covariance pairs, are not sensible to run here since the model uncertainty on *b*_1_, *b*_2_ and *β* is not very compatible with this prior form. We also find the IBR partition using the Bayes partition for the effective RLPP, which merges uncertainty in *θ* and *ρ*. In particular, we compute the partition error for all partitions of the images from Eq (11), and choose the partition with minimal partition error. Note that Eq (11) is found using the natural cost function in Eq (10), and the posterior partition probabilities in Eq (9), which is based on posterior label function probabilities that may be computed exactly using a discretized version of Eq (27). Recall *f*(**x**|*y*, *ρ*, *θ*) is assumed Gaussian with means given by Eq (25), covariances given by Eq (26), and appropriate values for *b*_1_, *b*_2_ and *β* depending on *y*, *ρ* and *θ*. It is possible to list all partitions and compute the partition errors exactly when *n* = 10 and *l* = 2. Again, we did not test MCBR and minimax robust clusterers owing to their high computational cost.

**Fig 5:**
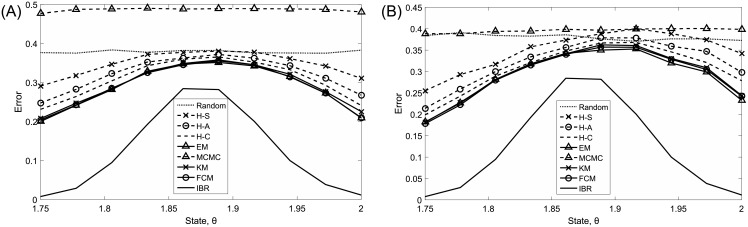
Average cluster mismatch error as a function of the state, *θ*, in the granular imaging example. (A) *n*_1_ = *n*_2_ = 5. (B) *n*_1_ = 6 and *n*_2_ = 4.

Fig 5 shows the approximate clustering error for all algorithms with respect to *θ*, computed using the average cluster mismatch error over 500 sets of images for each *θ*. Part A shows results when *n*_1_ = *n*_2_ = 5, and part B shows results when *n*_1_ = 6 and *n*_2_ = 4. In both parts A and B, the IBR clusterer performs much better than all classical algorithms across all states. Note that the IBR clusterer makes “incorrect” Gaussian modeling assumptions, but that the Gaussianity assumption and the analytically computed mean and covariance for each cluster become more accurate as the number of grains increases. Under all algorithms there is a significant variation in performance, which deteriorates when *θ* ≈ 1.8750. This corresponds to the case where *β*_1_ = *β*_2_, i.e., the case where the classes are most similar. Among all classical algorithms, the EM algorithm is usually the best, followed by FCM and KM, which have very similar performance. In some cases in Fig 5, the performance of hierarchical clustering with single linkage is worse than Random. As seen in the “Robust Clustering Under Gaussian RLPPs” section, MCMC with incorrect priors and small samples again has very poor performance. These graphs are summarized in Table 2, which shows the approximate average clustering error for each algorithm over all states and iterations. Finally, note that performance is similar between equal and unequal cluster size for all algorithms.

**Table 2:** Average cluster mismatch error over 5, 000 iterations in the granular imaging example.

*n*_1_, *n*_2_	IBR	FCM	KM	MCMC	EM	H-C	H-A	H-S	Random
5, 5	0.1239	0.2899	0.2938	0.4858	0.2890	0.3086	0.3223	0.3477	0.3786
6, 4	0.1351	0.2924	0.2952	0.3956	0.2904	0.3079	0.3224	0.3488	0.3799

Since our focus is on robust clustering theory rather than image processing, in particular, the interplay between clustering optimization and the structure of prior knowledge, we have implemented a model setting based on Eq (23); nevertheless, before concluding this section, we believe a few comments concerning the effect of deviations from the model assumptions on the asymptotic granulometric moments are warranted.

The grain model of Eq (23) has been used in numerous studies of granulometric filtering and asymptotic moment analysis. Three issues regarding robustness of the theory to deviations from model assumptions have been addressed in [32]: (1) assuming a certain sizing distribution when in fact the random set satisfies a different sizing distribution, (2) using erroneous parameters for the sizing distribution, and (3) prior segmentation when there is modest overlapping.

For instance, the effect of erroneous gamma(*α*, *β*) sizing was analytically quantified with respect to misclassification error. Perhaps more importantly, the effect of watershed segmentation to separate overlapping grains prior to moment analysis was quantified by establishing lower and upper bounds on the actual *k*th granulometric moments when there are multiple grain primitives. One could reconsider the entire clustering analysis relative to these bounds; however, given the complexity of the bounds, this would involve a complicated mathematical study that would lead us far afield. The bounds are quite tight when grain overlapping is minor, as it is with a properly prepared emulsion.

Finally, as in all asymptotic granulometric theory, grain orientation is assumed fixed and not subject to rotation. The assumption is that each grain can be canonically rotated so that triangles have a horizontal base and for rods the shorter side forms the base, as assumed in the model. Robustness relative to imperfect rotation normalization has not been studied analytically. In fact, in digital image processing, rotation can cause problems for triangles and rectangles when edge detection is inaccurate, which is troublesome when there is low pixel resolution, a situation that is much less problematic today than when the basic granulometric theory was developed twenty years ago.

## Conclusion

We have extended the theories of robust filtering and classification to clustering and developed new theory showing that optimal Bayesian robust clustering can be viewed as two equivalent optimization problems, one based on a parameterized uncertainty class of RLPPs and the other on a single effective RLPP that absorbs all parameters in the model. Thus, one can first focus on modeling the uncertainties and then focus on finding the Bayes clusterer (or a good approximation) for the effective model.

The proposed paradigm for robust clustering is distinct from all other clustering methods in that it is fully model-based, can account for all prior knowledge and sources of uncertainty, and is optimal relative to clustering error. A key part of the paradigm involves justifying the modeling assumptions. In cases where the modeling assumptions can be justified, like in our granular imaging example where we developed new theory on the asymptotic joint normality and moments of our extracted features, we now have a very powerful theory for optimal robust clustering. Furthermore, since the Bayes and IBR clusterers employ powerful optimization directly with respect to clustering error (or clustering risk if used with specialized cost functions), under small to moderate imperfections of the assumed model they often continue to outperform many principled optimization-based methods. For instance, although our implementations of the EM, MCMC and IBR algorithms all assume Gaussian mixture models, EM and MCMC do not always perform as well as IBR because: (1) they focus on estimating the means and covariances instead of minimizing error, (2) they are often implemented without available prior knowledge.

IBR clustering is useful in a wide range of applications, particularly in clustering problems where the underlying data generation process is unknown, but can be theoretically constrained or partially described using scientific knowledge. Our granular imaging application is an excellent example, where we use a theorem that justifies Gaussianity and constrain parameters of the sizing distributions to train an IBR clusterer that far outperforms other data-driven methods. IBR clustering makes distributional assumptions that are expected to improve performance when correct, but may also degrade performance when grossly incorrect. Although we have defined and characterized IBR clustering for general applications here, in specific applications prior construction and prior validation are critically important steps.

The IBR clusterer (and Bayes clusterer) can only be implemented under small samples with up to 20 or so points; when *n* is large approximations of the IBR clusterer are available. Near optimal performance is possible with reasonable run time, and by tweaking the approximation algorithm one can make a trade-off between performance and run time. Suboptimal methods inspired by the optimal equations for the Bayes clusterer under Gaussian models (e.g., Suboptimal Pseed Fast) presented in [1] have good performance and competitive computation time with point sets of size up to 10, 000. Whether the exact or approximate IBR clusterer is used, increasing dimensionality also increases computation time, but to a much smaller extent; here we have implemented IBR clustering on datasets with up to 1, 000 features. Nevertheless, new suboptimal algorithms and methods to address computation remain important topics for further research. Since our objective here has been to develop a basic framework for robust clustering, we have focused on examples with relatively small point sets, implemented optimal algorithms whenever possible, and strongly favored better performing suboptimal algorithms at the expense of run time. We aim to continue developing improved algorithms for Bayes and IBR clustering in future work.

## Supporting information

S1 File Minimal data set for the Gaussian example. This file contains the number of mismatches (integer count of errors) between the correct label and label output by each algorithm (mismatch error is minimized over all sets of labels that induce the same partitions) in each iteration, and the run time for each algorithm in each iteration, which are used to generate Fig 2 and Table 1.
(ZIP)

S2 File Minimal data set for the granular imaging example. This file contains the number of mismatches between the correct label and label output by each algorithm in each iteration, which are used to generate Fig 5 and Table 2. This file also contains raw data and MATLAB code used to generate Fig 4.
(ZIP)

S1 Appendix Granulometry theorems. This file contains three granular imaging theorems that justify modeling assumptions like normality used by the IBR clusterer in The granular imaging example.
(PDF)
