# Supplementary material for: Optimal clustering under uncertainty
Source: PLoS One. 2018 Oct 2;13(10):e0204627. doi: 10.1371/journal.pone.0204627 (PMC6168142; doi:10.1371/journal.pone.0204627)
Supplement: S1 Appendix — This file contains three granular imaging theorems that justify modeling assumptions like normality used by the IBR clusterer in The granular imaging example. (PDF) [file pone.0204627.s003.pdf]

Optimal Clustering under Uncertainty

Lori A. Dalton[1*], Marco E. Benalcázar[2], Edward R. Dougherty[3]

1 Department of Electrical and Computer Engineering, The Ohio State University, Columbus, OH, USA
2 Departamento de Informática y Ciencias de la Computación, Facultad de Ingeniería de Sistemas, Escuela Politécnica Nacional, Quito, Ecuador
3 Department of Electrical and Computer Engineering, Texas A&M University, College Station, TX, USA

* dalton.lori@outlook.com

S1 Appendix

Here, we justify modeling assumptions like normality used by the IBR clusterer in the granular imaging example. The following theorems, originally proved in [1] and [2], provide exact expressions for granulometric moments as a function of the grain radii. They state that any finite length vector of granulometric moments from a single structuring element is asymptotically normal, and provide analytic expressions for the asymptotic mean and variance of moments. The covariance of moments is available in [2].

Theorem 1. *Let I be modeled as in Eq. (23) of the main manuscript. For the granulometry $\{I \circ tB\}$ generated by a convex, compact structuring element B, and for $k \geq 1$,*

$$\mu^{(k)}(I, B) = \frac{u}{v} \equiv H(u, v), \tag{1}$$

where

$$u = \frac{1}{N} \sum_{i=1}^{m} \sum_{j=1}^{N_i} \mu^{(k)}(A_i, B) \nu[A_i] r_{ij}^{k+2}, \tag{2}$$

$$v = \frac{1}{N} \sum_{i=1}^{m} \sum_{j=1}^{N_i} \nu[A_i] r_{ij}^{2}, \tag{3}$$

$\nu[A_i]$ is the volume of A_i, and $\mu^{(k)}(A_i, B)$ is the kth moment of A_i under structuring element B. Moreover, suppose:

1. *The proportions $b_i = N_i/N$ are known and fixed.*
2. *The r_{ij} are independent, $r_{i1}, \ldots, r_{iN_i}$ are identically distributed, and every r_{ij} has finite moments up to at least order $k+2$.*
3. *There exist c and $t > 0$ such that $H \leq cN^t$ for $N > 1$.*
4. *H has first and second derivatives, with bounded second derivatives in a neighborhood of $(E[u], E[v])$.*

Then the distribution of H is asymptotically normal as $N \to \infty$ with mean and variance given by

$$E[H] = H(E[u], E[v]) + O(N^{-1}) \tag{4}$$

and

$$\begin{aligned} Var[H] &= (\tfrac{\partial H}{\partial u}(E[u], E[v]))^2 Var[u] \\ &+ 2\tfrac{\partial H}{\partial u}(E[u], E[v])\tfrac{\partial H}{\partial v}(E[u], E[v]) Cov[u, v] \\ &+ (\tfrac{\partial H}{\partial v}(E[u], E[v]))^2 Var[v] + O(N^{-3/2}). \end{aligned} \tag{5}$$

Theorem 2. *Under the conditions of Theorem 1, any finite set of granulometric moments is asymptotically jointly normal.*

Theorems 1 and 2 are not sufficient to guarantee the asymptotic joint normality of $\mathbf{x}$ or $\mathbf{z}$, or to obtain their asymptotic moments, because these vectors contain moments from multiple structuring elements. The following Theorem, originally proved in [3], guarantees asymptotic joint normality and provides analytic expressions for the asymptotic mean and covariance of granulometric moments under multiple primitives and multiple structuring elements. It can be shown that these moments are consistent with Eqs. (4) and (5).

Theorem 3. *Let I be modeled as in Eq. (23) of the main manuscript with $m = 2$ primitives, let $b_1 = N_1/N$ and $b_2 = N_2/N$ be known and fixed, and let the radii r_{ij} be independent such that $r_{i1}, \ldots, r_{iN_i}$ are identically distributed and $E[r_{ij}^k] = \gamma_{ik}\beta^k$ for $k = 2, 3, 4$. Let $\mathbf{x} = M^{-1}\mathbf{z}$ be a vector of linearly transformed first and second order granulometric moments under arbitrary structuring elements, B_1 and B_2. Then $\mathbf{x}$ is asymptotically jointly normal with mean vector*

$$\frac{1}{b_1\gamma_{12} + b_2\gamma_{22}} \begin{bmatrix} b_1\gamma_{13}\beta & b_2\gamma_{23}\beta & b_1\gamma_{14}\beta^2 & b_2\gamma_{24}\beta^2 \end{bmatrix}^T \tag{6}$$

and covariance matrix

$$\frac{1}{N(b_1\gamma_{12} + b_2\gamma_{22})^4} \begin{bmatrix} A_{11}\beta^2 & A_{12}\beta^3 \\ A_{21}\beta^3 & A_{22}\beta^4 \end{bmatrix}, \tag{7}$$

where

$$\begin{aligned} A_{ij} &= \begin{bmatrix} (b_1)^2(b_1 B_{ij1}^{11} + b_2 B_{ij1}^{12}) & b_1 b_2(b_1 B_{ij1}^{21} + b_2 B_{ij1}^{22}) \\ b_1 b_2(b_1 B_{ij2}^{11} + b_2 B_{ij2}^{12}) & (b_2)^2(b_1 B_{ij2}^{21} + b_2 B_{ij2}^{22}) \end{bmatrix} \\ &+ \begin{bmatrix} b_1(b_1\gamma_{12} + b_2\gamma_{22})^2 C_{ij1} & 0 \\ 0 & b_2(b_1\gamma_{12} + b_2\gamma_{22})^2 C_{ij2} \end{bmatrix}, \\ B_{ijk}^{lp} &= C_{00p}\gamma_{k(i+2)}\gamma_{l(j+2)} \\ &- \gamma_{p2}\gamma_{k(i+2)} C_{0jl} - \gamma_{p2} C_{i0k}\gamma_{l(j+2)}, \\ C_{ijk} &= \gamma_{k(i+j+4)} - \gamma_{k(i+2)}\gamma_{k(j+2)}. \end{aligned}$$

References

1. Sand F, Dougherty ER. Asymptotic Granulometric Mixing Theorem: Morphological Estimation of Sizing Parameters and Mixture Proportions. Pattern Recognition. 1998;31:53–61.

2. Sivakumar K, Balagurunathan Y, Dougherty ER. Asymptotic joint normality of the granulometric moments. Pattern Recognition Letters. 2001;22:1537–1543.

3. Dalton LA. Joint asymptotic normality of granulometric moments under multiple structuring elements. Pattern Recognition Letters. 2018;111:80–86.
